# Comparison of primary health care services between urban and rural settings after the introduction of the first urban health centre in Vyronas, Greece

**DOI:** 10.1186/1472-6963-8-124

**Published:** 2008-06-09

**Authors:** Anargiros Mariolis, Constantinos Mihas, Alevizos Alevizos, Theodoros Mariolis-Sapsakos, Konstantinos Marayiannis, Marek Papathanasiou, Vassilios Gizlis, Dimitris Karanasios, Bodossakis Merkouris

**Affiliations:** 1 Health Centre of Vyronas, Athens, Greece; 2 Health Centre of Nea Madytos, Thessaloniki, Greece; 3 Greek Association of General Practitioners (ELEGEIA), Greece

## Abstract

**Background:**

Discrepancies in primary health care (PHC) services between urban and rural settings have already been studied in many countries; however, limited information exists regarding countries, such as Greece, where public Health Centres dedicated to primary care have not been in existence in major cities. The objective of this study was to evaluate points of divergence or convergence between an urban and a rural health centre, in an attempt to underline challenges faced by the introduction of urban health centres in Greece.

**Methods:**

A cross-sectional analysis was conducted in the Health Centre of Vyronas, Athens, Greece and in the Health Centre of Nea (New) Madytos, Thessaloniki Prefecture, Greece between February 2004 and February 2006. The profile of the population seeking care, as well as data on the services provided were collected and compared. In addition, the reason for choosing each primary health care unit was also recorded.

**Results:**

More patients visited the urban centre (145415 vs. 112513), while the pattern of services utilized by the citizens differed significantly (p < 0.001) between the two Health Centres. The frequency of diagnoses made according to ICPC-2 was not similar in the two Health Centres (p < 0.001). The three most frequent reasons for the adults choosing the Health Centre for their problem were low waiting time, proximity to residence and satisfaction with the services provided in previous visits in Vyronas.

**Conclusion:**

The results of this study highlight the significant differences regarding PHC services utilization between an urban and a rural population. Urban citizens seem to have different health needs and reasons for choosing a PHC unit than residents of the Greek countryside. Proximity to health services and the public character of the urban health centre seem to be its main advantages.

## Background

Primary health care (PHC) is mainly provided by General Practitioners – Family Physicians (GPs) in developed countries [1-3] This is not the case in developing countries, where many doctors who are not GPs work in PHC [4]. In Greece, the National Health System (NHS) is organized in a way that local PHC units (known as Health Centres) are responsible for PHC in their region [5]. More than half the medical personnel working in these Centres are GPs. In rural areas, this type of organization seems well established, since 212 Health Centres exist; on the other hand, in major cities, such as Athens or Thessaloniki, there was not a single primary care dedicated unit till now. In metropolitan areas, PHC is mainly provided by the Social Insurance Institute (SII) [6] and to a lesser degree by private clinics. However, SII covers only those in dependent employment or those who offer full-time or part-time personal labour on commissioned work agreements and are not insured with any other insurance agency (i.e. about 50% of the Greek population) [7]. What is more, the health units of SII should be characterized as secondary care units, as they are staffed with doctors of all specialties. Problems frequently arise in these units, since they do not offer 24-7 coverage, patients are self-referred and there is no continuity of care, as the physicians change posts very often in search of better remuneration.

Even though rural – urban differences in access and usage of PHC have already been appraised in several studies, they are usually documented in countries with a health system providing PHC in the metropolitan areas [8-20]. According to those studies, rural residents were older [8], had more financial problems (lower per capita income and higher poverty rate) [9], were more likely to be uninsured [10], were less educated [11] and faced more obstacles when trying to access health services providers, such as longer travel distances and lack of transportation [12]. Additionally, the scarcity of hospitals and physicians in rural areas may influence the quality of PHC by limiting the variety of health services provided [13].

The rationale for conducting this study was the peculiarity of the Greek NHS. In the context of an already well established and organized rural-oriented PHC system functioning for more than 20 years, urban PHC has only recently been introduced, with the experimental and pilot operation of the first urban health centre in the country, the Health Centre of Vyronas (HCOV) [14]. Although the use of PHC in a Greek rural setting has been already recorded and compared with a European analogue [15], the differences between an urban and rural PHC unit have not been investigated, especially in a country where PHC is rudimentary in metropolitan areas. In major cities, the Greek healthcare system follows the trend towards super-specialized and inter-hospital medicine, made necessary by the explosion of new knowledge in the field of bio-medical research and the attractiveness due to its better 'market value' [16]. The low status of General Practice in our country is also reflected upon the low proportion (4.3%) of undergraduate medical students in their senior year willing to choose GP as a career choice [17].

The objective of this study was to evaluate differences in the utilization of rural and urban PHC services, as well as to compare the patients' reasons for visit and diagnoses patterns, since they may differ from those in an urban or a rural setting in other European countries. In addition, this comparison may allow us to appraise the pioneering implementation of dedicated Primary Health Centres in a Greek urban area, given the particularities of Greek NHS, as mentioned above.

## Methods

The study was based on a cross-sectional analysis of data collected in the Health Centre of Vyronas, Athens, Greece (HCOV) and the Health Centre of Nea (New) Madytos, Thessaloniki Prefecture (HCNM) between February 2004 and February 2006. The HCOV is located in Vyronas, a densely populated district in Athens metropolitan area, covering at least 61102 citizens (according to the 2001 census, not calculating those from neighbouring municipalities). The HCOV was established in 2004 due to the efforts of a group of GPs and was afterwards integrated in the National Health System. All doctors of HCOV are GPs, both specialized and residents. Furthermore, the HCOV is staffed by nurses, midwives, health visitors, administrative personnel and research associates (biostatistician and computer programmer). Following a basic plan for Health Centres across Greece, HCOV has an emergency department and a chronic diseases – follow-up clinic. There is also a paediatric and an ambulatory care department. In addition to this basic scheme, HCOV incorporates also a health promotion – preventive medicine medical team responsible for various health promotion and educational programs in schools and special social groups of its prefecture as well as a biostatistics office. Moreover, HCOV is an educational centre for pre-graduate medical students and after-graduate nursing school students, health visitors and General Practice/Family Medicine residents.

The HCNM is located in Nea Madytos, 60 km east of Thessaloniki, Macedonia, Northern Greece, providing primary medical care for a mainly agricultural population of at least 3456 citizens (taking into account the municipality it belongs to). In addition, it is estimated that approximately 10000 people living in neighbouring areas are also covered by the HCNM. The staff of the HCNM does not differ from that in HCOV, except for the presence of doctors of other medical specialties (internists, cardiologists, surgeons) and the lack of research associates (Table 1). It should also be noted that, since Nea Madytos is a summer resort, the population covered rises up to almost 60000 during the summer period. Additionally, due to the small distance between HCNM and the international highway, many travellers are visiting it for various reasons as they pass through. All patients seeking care in the HCNM, regardless of the specialty of the doctor, were included in the study.

**Table 1:** Staffing and Services provided by HCOV and HCNM

	HCOV	HCNM
**Staff**		

**Medical**		
General Practitioners (Specialized)	3	6
General Practitioners (Residents)	9	5
Internists	0	1
Cardiologists	0	1
Surgeons	0	1
Psychiatrists	0	1
Ophthalmologists	0	1
Paediatricians	0	2
**Nurses**	7	8
**Midwives**	1	2
**Health visitors**	4	2
**Administrative**	4	3
**Research associates**	2	0
		
**Services**		

Emergency department	1	2
Chronic diseases – follow up clinic	2	3
Paediatric department	1	1
Surgery department	0	1
Ophthalmology department	0	1
Psychiatry office	0	1
Ambulatory care department	1	0
Health promotion – Preventive medicine department	1	0
Biostatistics office	1	0
Science Education office	1	0

An electronic form constructed by the authors in Microsoft^® ^Access 2003 was used in the HCOV departments, asking participating doctors to fill in information from each patient, including patient demographics, reason(s) for visit and diagnosis. Additionally, the patient answered to a multiple-choice questionnaire regarding the reason for choosing the Health Centre instead of other PHC facilities (if any, like private clinics, SII clinics, hospitals). In this form, the doctors also recorded other data, such as medical procedures performed and medications prescribed (not incorporated in the analysis). The same form was also used in HCNM. For each categorical variable (demographics, reason for visit, diagnosis, reason for choosing), the form was providing a selection from a special list. Reasons for visit and diagnoses were organized according to International Classification of Primary Care, 2nd edition [18]. The form allowed the physician to register more than one reason for visit and diagnosis. If the patient was a child, the responses were given by their parent or grandparent, while if the patient was unable to give reliable information due to mental problems or physical inability, such as major trauma or coma, data were collected from their spouse or caregiver. The part of the questionnaires administered regarding demographic characteristics and reason for choosing the Health Centre was completed by the patient and was given to an administrative officer who was responsible for this part of the data collection. For home visits, physicians carried a Personal Digital Assistant (PDA) in order to record the patients' data on the spot. At the end of each week, data were collected and analyzed for any mistakes made from the physicians in staff meetings. The urban participants were also invited to report the PHC services provider they had been visiting before the establishment of HCOV. Prior to the study, a random sample of 102 patients in HCOV and 92 patients in HCNM was used for pilot reasons, in order to detect any problems that might be encountered during the data collection procedure and validate our questionnaire. In addition, the participants of the pilot study described the reasons for choosing the health centre in open format questionnaire, allowing us to construct the closed format questionnaire that was delivered during the main study.

The physicians who participated were restricted to GPs, as they are the overwhelming majority of the PHC physicians in Greece. The above had to attend a 5-day educational course conducted by the authors regarding the proper use of the electronic system.

The study protocol complies with the Declaration of Helsinki, was approved by our institutional Ethics Committee and all participants gave written informed consent.

### Statistical analysis

The data collected were transferred to an electronic database. The results were classified and reviewed according to age groups which were selected based on the labour force participation and in order to have a homogenous distribution across groups. Therefore, the following age groups were used: a) Children <15 years old to include paediatric health problems, b) Adults 15–64 years old to reflect those who are engaged in the labour force, c) Elderly more or equal than 65 years old to reflect those who are retired and suffer from common health problems of the third age.

The following variables were used as stratification factors: Age (9 decades, 9 strata), gender (males and females, 2 strata), marital status (singles, married, divorced, widowers/s, 4 strata), educational status (tertiary, secondary and primary level, analphabetic, 4 strata), nationality (Greek, other, 2 strata), financial status (≤€15,000, > €15,000, 2 strata). The relative frequencies of the stratification factors were pre-defined based on the data from the national census of 2001 [7]. As a result, the two new samples had similar distribution of the aforementioned parameters, adjusting for their potential effect on the attributes examined in our study. The power analysis showed that a number of 5902 participants in each group was adequate in order to detect real proportion differences greater than 0.03, achieving power 90% at a significance level of 0.05.

For the comparison of distribution of gender, age, PHC services, diagnoses and frequencies of special groups between the two health centres, the Pearson chi-square statistic was used. In order to estimate any potential monthly trend of the patients who visited the two health centres in 2005 we calculated the Pearson chi-square for trend statistic. The level of significance was set at p < 0.05. Data were analyzed using STATA™ (Version 9.0, Stata Corporation, College Station, TX 77845, USA).

## Results

### Demographics

Details regarding demographic characteristics of the populations living in Vyronas and Nea Madytos are shown in Table 2.

**Table 2:** Demographic characteristics of populations of Vyronas and Nea Madytos

	**Vyronas (HCOV)**		**Nea Madytos greater area (HCNM)**	
		
	**N**	**%**	**N**	**%**
**Total population**	61102		13348	
**Gender**				
**Men**	29241	47.86%	6770	50.72%
**Women**	31861	52.14%	6578	49.28%
**Age groups**				
**0–4**	2740	4.48%	666	4.99%
**5–9**	2754	4.51%	668	5.01%
**10–14**	2933	4.80%	655	4.91%
**15–19**	3804	6.23%	785	5.88%
**20–24**	4889	8.00%	959	7.18%
**25–29**	5141	8.41%	940	7.04%
**30–34**	5335	8.73%	977	7.32%
**35–39**	4701	7.69%	802	6.01%
**40–44**	4696	7.68%	828	6.21%
**45–49**	4319	7.07%	781	5.85%
**50–54**	4153	6.80%	823	6.17%
**55–59**	3161	5.17%	800	6.00%
**60–64**	3286	5.38%	1041	7.80%
**65–69**	3036	4.97%	985	7.38%
**70–74**	2688	4.40%	861	6.45%
**75–79**	1713	2.80%	415	3.11%
**80–84**	1011	1.65%	194	1.45%
**85+**	743	1.22%	165	1.23%
**Marital status**				
**Singles**	24960	40.85%	4577	34.29%
**Married**	28810	47.15%	7490	56.11%
**Widows/Widowers**	4354	7.13%	1056	7.91%
**Divorced**	2978	4.87%	225	1.69%
**Educational level**				
**Master or PhD**	1053	1.72%	141	1.06%
**University/College graduates**	10501	17.19%	1930	14.46%
**High School graduates**	23439	38.36%	4450	33.34%
**Elementary school graduates or still studying**	25108	41.09%	6565	49.18%
**Analphabetic**	1000	1.64%	262	1.96%
**Nationality**				
**Greek**	55114	90.20%	12524	93.82%
**Other**	5988	9.80%	824	6.18%
**Family yearly income**				
**≤ €15,000**	19760	32.34%	6045	45.29%
**>€15,000**	41342	67.66%	7303	54.71%

Even though the population covered by the HCNM is growing during the summer season for reasons mentioned before, we had to use population data according to the national census in order to obtain reliable information about the distribution of age. The HCOV is responsible for a population that is about 4 times larger than that of the HCNM. The gender distribution did not differ (p = 0.581) between the two populations and was similar to the national population [7]. However, the distribution of age groups was significantly different between the two areas, since a trend towards older age was described in New Madytos (>= 65 years old 21.42% vs 13.34% in Vyronas, p < 0.001). As a consequence, children 0–14 years old formed a larger segment of the urban population compared with the rural area (22.34% for Vyronas vs. 16.53% for Nea Madytos).

Based on the distribution of PHC services provided by both health centres (Table 3), there were more patients visiting the HCOV during the time of the study than the HCNM (145415 vs. 112513). The patients per population per year and contacts per population per year ratios were higher at the HCNM than at the HCOV (3.64 vs. 1.19, p < 0.001 and 6.52 vs. 3.14, p < 0.001, respectively). The average patient visited more frequently the HCOV than the HCNM, as it is derived by the higher contacts per patient per year ratio (2.64 vs. 1.79, p < 0.001). The pattern of PHC services utilized by the citizens differed significantly (p < 0.001) between the two Health Centres. The referral rates to secondary or tertiary care hospitals were significantly lower in HCOV in all age groups, ranging from 1.06% to 4.53% in children, compared to the HCNM (referral rate: 4.00% to 10.02%, p < 0.001).

**Table 3:** Distribution of Primary Health Care Services provided by HCOV and HCNM and index of utilization of health services during 2004–2006.

		**HCOV**		**HCNM**		
			
**Department**	**Age group**	**N**	**%**	**N**	**%**	**p-value**
	**0–14**					
						
Pediatric Clinic		28945	91.18	12013	89.41	
Vaccinations		2701	8.51	1411	10.50	<0.001
Home visits		99	0.31	12	0.09	
Total		31745	100.00	13436	100.00	
						
Referrals to secondary or tertiary care hospitals		1311	4.53	1204	10.02	<0.001

	**15–64**					
						
Chronic diseases – Follow up		27822	57.81	37050	72.66	
Emergency Room		19432	40.38	12893	25.28	
Vaccinations		722	1.50	1013	1.99	<0.001
Home visits		147	0.31	38	1.48	
Total		48123	100.00	50994	100.00	
						
Referrals to secondary or tertiary care hospitals		654	1.38	2563	5.13	<0.001

	**>= 65**					
						
Chronic diseases – Follow up		56323	85.93	35365	73.55	
Emergency Room		7799	11.90	11341	23.59	
Vaccinations		787	1.20	1299	2.70	<0.001
Home visits		638	0.97	78	0.16	
Total		65547	100.00	48083	100.00	
						
Referrals to secondary or tertiary care hospitals		677	1.06	1867	4.00	<0.001

	**Total**					
						
Chronic diseases – Follow up		84145	57.87	72415	64.36	
Emergency Room		27231	18.73	24234	21.54	
Pediatric Clinic		28945	19.91	12013	10.68	
Vaccinations		4210	2.90	3723	3.31	<0.001
Home visits		884	0.61	128	0.11	
Total		145415	100.00	112513	100.00	
						

Referrals to secondary or tertiary care hospitals		2642	2.37	5634	5.83	<0.001

						

Patients per population per year		1.19		3.64		<0.001
Contacts per population per year		3.14		6.52		<0.001
Contacts per patient per year		2.64		1.79		<0.001

The results of comparison between the two subgroups that were constructed *post hoc *and adjusted for main demographic confounders are presented in Table 4. According to this analysis, citizens of Nea Madytos visited more frequently the chronic diseases department than those of Vyronas (64.23% vs. 52.51%). On the other hand, more patients were admitted in the Emergency Department of HCOV than in that of HCNM (21.26% vs. 17.78% respectively). In addition, both paediatric clinic use and home visits differed between the two Health Centres (HCOV: 13.29%, 5.62% vs. HCNM: 9.53%, 2.03%, respectively). The total distribution of PHC services used in the two Health Centres differed significantly (p < 0.001). The adjusted referral rate was significantly higher in the HCNM than in the HCOV (6.23% vs. 2.41%, p < 0.001), while the same patients seemed to visit more frequently the HCOV than the HCNM (Contacts per patient per year ratio: 3.04 vs. 1.67, respectively, p < 0.001).

**Table 4:** Main characteristics of adjusted* samples of HCOV and HCNM

**Department used**					
	
	**HCOV**		**HCNM**		
			
	**N**	**%**	**N**	**%**	**p-value**
Chronic diseases – Follow up	3099	52.51%	3791	64.23%	
Emergency Room	1255	21.26%	1049	17.78%	
Pediatric Clinic	784	13.29%	562	9.53%	
Vaccinations	432	7.32%	379	6.43%	<0.001
Home visits	332	5.62%	120	2.03%	
**Total**	**5902**	**100.00%**	**5902**	**100.00%**	

**Referral rate**					
Referrals to secondary or tertiary care hospitals	142	2.41%	368	6.23%	<0.001

					

**Contacts per patient per year**					
Contacts per patient per year (ratio)	3.04		1.67		<0.001

					

**Top 5 reasons for visit (ICPC-2)**					
		
	**Relative frequency (%)**	**Rank**	**Relative frequency (%)**	**Rank**	
		
Cough	10.04	1	7.16	1	
Fear of hypertension	9.32	2	6.89	2	
Headache	6.73	3	4.26	6	
Fever	5.38	4	6.42	3	
Chest symptom	5.14	5	5.62	4	

					

**Top 5 reasons for choosing PHC unit**					
		
	**Relative frequency (%)**	**Rank**	**Relative frequency (%)**	**Rank**	
		
It is close to my home	52.67	1	34.21	2	
Was satisfied of previous visit	51.47	2	23.45	4	
Low time to wait	25.49	3	10.46	7	
It is free	23.56	4	24.62	3	
It was the only one open around	13.62	5	40.57	1	

* Adjusted for gender, age, financial income, educational status, marital status and nationality.

**Figure 1:**
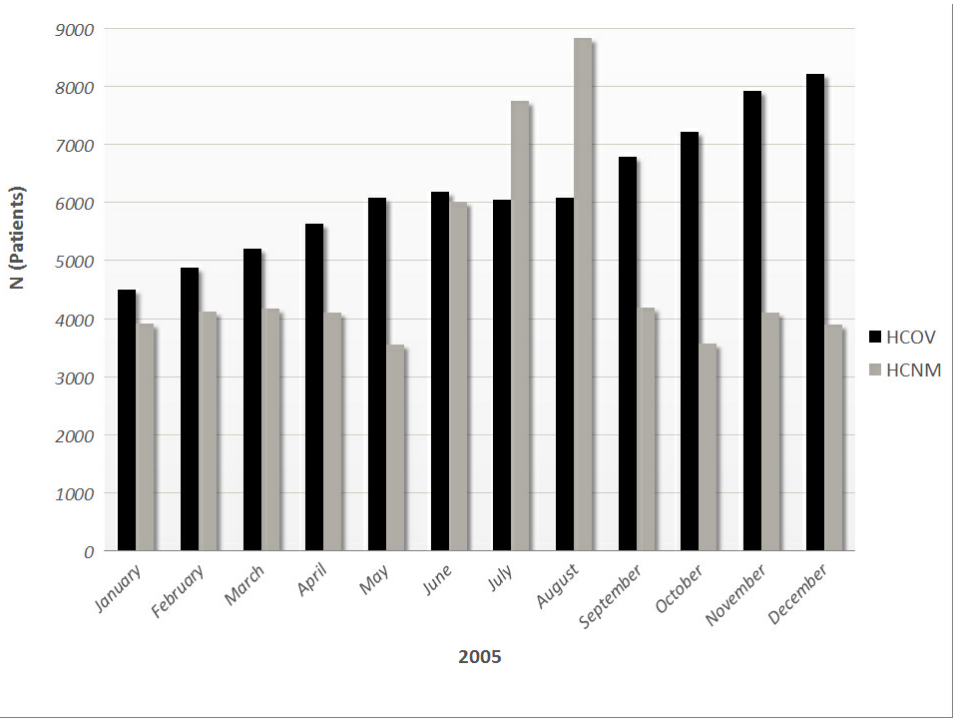
Monthly distribution of PHC users during 2005 in HCOV and HCNM.

During 2005, the number of citizens who utilized the PHC services of the HCOV steadily increased (p < 0.001). A peak was recorded during summer months in the HCNM followed by a fall to the first 6 months levels after September (Figure 1). Private medical offices (45.12%), followed by public secondary or tertiary care hospitals (24.78%), private hospitals (15.84%) and pharmacies (7.35%) were the PHC providers the respondents used before the establishment of HCOV.

### Diagnoses, reasons for visit and for choosing a Health Centre

In the age group 0–14 years, cough and throat symptoms were the two most common in both HCOV and HCNM, while cough, headache and back symptoms dominated in the 15–64 years group in the HCOV (Table 5). On the other hand, the top three symptoms in the same age group in the HCNM were back symptoms, muscle pain and skin rash. The elderly citizens (>= 65 years old) were more likely to visit HCOV complaining for hypertension, chest and joint symptoms. Similar results were also reported in the HCNM.

**Table 5:** Distribution of top 10 reasons for visit according to ICPC-2 by Health Center and age categories, 2004–2006

	**HCOV**		**HCNM**	
		
**Reason for visit**	**Relative frequency (%)**	**Rank**	**Relative frequency (%)**	**Rank**
	**Age group: 0–14**			

Cough	10.12	1	9.45	1
Throat symptom/complaint	8.72	2	8.34	2
Headache	7.70	3	5.56	6
Fever	7.65	4	7.45	3
Ear pain/earache	4.32	5	4.76	7
Skin rash localized	4.19	6	7.17	5
Muscle pain	4.12	7	7.23	4
Medical examination for school etc.	4.01	8	4.12	8
Abdominal pain epigastric	3.03	9	1.85	12
Urinary symptoms	1.89	10	0.65	13
				

**Age group: 15–64**				

Cough	9.82	1	7.31	5
Headache	7.45	2	4.33	6
Back symptoms	6.23	3	10.12	1
Urinary symptoms	5.14	4	2.02	12
Fear of hypertension	5.12	5	2.81	11
Muscle pain	4.96	6	7.73	2
Skin rash localized	4.79	7	7.61	3
Chest symptom	4.12	8	4.31	7
Fear of endocrine disorder	3.56	9	2.92	9
Joint symptom	3.44	10	7.37	4
				

**Age group: >= 65**				

Fear of hypertension	15.23	1	13.47	1
Chest symptom	8.92	2	8.41	5
Joint symptoms	8.76	3	11.56	2
Cough	7.54	4	8.38	6
Fever	6.79	5	7.22	7
Limited function/disability	6.20	6	9.92	3
Fear of endocrine disorder	5.78	7	6.13	8
Abdominal pain epigastric	5.54	8	2.23	12
Sleep disturbance	5.34	9	8.73	4
Shortness of breath/Dyspnoea	5.12	10	6.01	9

The frequency of diagnoses made according to 17 different clusters of classification based on the ICPC-2 is described in Table 6. As it is shown, the distribution of the diagnoses was not similar in the two Health Centres (p < 0.001), since the diseases of the cardiovascular system were the most frequent in HCOV, followed by diseases of the respiratory and musculoskeletal system. In the HCNM, the diseases of the respiratory system dominated (21.56%), followed by diseases of the cardiovascular and musculoskeletal system.

**Table 6:** Distribution of diagnoses and ranks by classification according to the ICPC-2 by Health Center, 2004–2006

	**HCOV**		**HCNM**	
		
**Diagnosis**	**Relative frequency (%)**	**Rank**	**Relative frequency (%)**	**Rank**
Diseases of the cardiovascular system	19.34	1	16.45	2
Diseases of the respiratory system	18.75	2	21.56	1
Diseases of the musculoskeletal system	9.78	3	11.67	3
General and unspecified conditions	9.46	4	10.02	4
Psychological disorders	6.82	5	6.15	5
Endocrine, metabolic and nutritional disorders	6.45	6	4.64	7
Social problems	5.63	7	4.23	8
Diseases of the digestive system	4.56	8	2.15	13
Neurological disorders	4.06	9	3.87	10
Eye and ear related problems	3.36	10	5.63	6
Diseases of the skin.	3.01	11	4.12	9
General infectious diseases	2.41	12	3.23	11
Urological disorders	1.9	13	2.6	12
Neoplasms	1.79	14	1.12	15
Diseases of the female and male genital systems	1.08	15	0.68	16
Pregnancy, Childbearing and Family Planning	0.82	16	0.21	17
Diseases of the blood, blood-forming organs and immune mechanism	0.78	17	1.67	14

p < 0.001

The relative frequency of people of other than Greek nationality using the services of HCOV was significantly higher than in HCNM (10.78% vs. 2.87%, p < 0.001) (Table 7). This pattern was similar in all age groups. Moreover, a 3.85% of the PHC users in Vyronas and 0.71% in Nea Madytos were not insured (p < 0.001).

**Table 7:** Profile of PHC users according to their nationality and insurance status

		**n**	**Relative frequency (%)**	**n**	**Relative frequency (%)**	**p-value**
				
	**Age group**	**HCOV**		**HCNM**		
	0–14	4956	15.61	883	6.57	<0.001
Foreigners	15–64	6845	14.22	1889	3.70	<0.001
	>-65	3877	5.91	462	0.96	<0.001
	
	**Total**	15678	10.78	3234	2.87	<0.001

	0–14	2782	8.76	391	2.91	<0.001
Not insured	15–64	2452	5.10	342	0.67	<0.001
	>-65	367	0.56	68	0.14	<0.001
	
	**Total**	5601	3.85	801	0.71	<0.001

The three most frequent reasons for the adults choosing the Health Centre for their problem were low waiting time, proximity to residence and satisfaction with the services provided in previous visits in Vyronas. This was not the case in Nea Madytos (Table 8). The importance of the Health Centre being the only choice for patients in a rural area was reflected by its high ranks among the reasons for choosing it in Nea Madytos while it was not so in Vyronas (5th to 7th rank).

**Table 8:** Reasons for choosing HCOV and HCNM during 2004–2006

	**HCOV**		**HCNM**	
		
**Reason for choosing Health Centre**	**Relative frequency (%)**	**Rank**	**Relative frequency (%)**	**Rank**
	**Age group: 0–14**			

It is close to my home	33.43	1	38.13	2
Was satisfied of previous visit	28.12	2	18.81	4
It is free	27.23	3	16.68	5
My personal doctor unavailable	16.91	4	3.13	8
Low time to wait	12.13	5	7.45	6
It was the only one open around	9.85	6	43.37	1
Don't know where else to go	5.56	7	19.39	3
Other/Don't answer	4.85	8	6.89	7
				

**Age group: 15–64**				

Was satisfied of previous visit	59.72	1	35.78	3
It is close to my home	47.65	2	18.89	5
Low time to wait	38.78	3	46.62	2
It is free	29.82	4	24.38	4
It was the only one open around	21.67	5	48.83	1
My personal doctor unavailable	8.81	6	3.44	8
Other/Don't answer	5.15	7	6.45	7
Don't know where else to go	2.11	8	12.12	6
				

**Age group: >= 65**				

It is close to my home	63.39	1	45.43	1
Was satisfied of previous visit	47.73	2	31.45	4
It is free	34.69	3	39.98	2
Low time to wait	28.11	4	15.34	5
Don't know where else to go	25.19	5	33.67	3
My personal doctor unavailable	16.74	6	4.22	8
It was the only one open around	14.34	7	12.48	6
Other/Don't answer	9.89	8	7.67	7

The top five reasons for visit were almost similar in the adjusted for gender, age financial income, educational status, marital status and nationality samples of both Health Centres (Table 4). Cough and fear of hypertension were the most prevalent (10.04% and 9.32% in HCOV, 7.16% and 6.89% in HCNM) followed by fever and chest symptoms. Headache was a more common symptom for those living in an urban area (6.73%) than in a rural one (4.26%). Proximity to home (52.67%), satisfaction with previous visit (51.47%) and shorter waiting time (25.49%) were the three most important reasons for choosing the urban HCOV. At the same time, the top three reasons for the rural HCNM were availability (40.57%), proximity to home (34.21%) and free services (24.62%) (Table 4).

## Discussion

The results of the study indicate that there are significant differences between urban and rural PHC provided by community health centres in Greece. Even though several studies had already underlined those differences [19,20], they were conducted in countries with a well-established PHC urban network. The uniqueness of this study is that it compared the use of PHC services between a well established rural and the almost experimental, first urban health centre in the country.

The urban population differed from the rural one in terms of age distribution, since rural residents were older. This finding was in accordance with other studies [8,19], justifying our choice to classify the populations in age groups and make two adjusted samples. A reason for this trend might be the higher unemployment rates in rural areas [21,22], pushing young people to immigrate to major cities in search of a job. After adjusting for several demographic characteristics, rural citizens seemed to use the chronic diseases department in their Health centre more frequently than those in the city. This finding may be explained by the shortage or lack of other medical services (less private medical offices, remote hospitals) in a rural area compared with an urban environment. The relatively great difference regarding use of paediatric services in the unadjusted samples is not so evident in our adjusted study groups; this could be attributed to the older age distribution among rural citizens and the larger prevalence of immigrants (younger age distribution) in the urban area, factors that were eliminated during the adjustment.

More patients per population per year visited the rural health centre. However, it was impossible to count the large amount of visitors and vacationers during the summer period. In an attempt to adjust for this confounding factor, we excluded the two 6 month periods from April to September for both years of our study after the main analysis. Nevertheless, the patients per population per 6 month period (October to March) ratio was still higher in the rural area (2.43 vs. 1.87, p < 0.001). A probable but not evident explanation for this difference might be that HCNM is the sole PHC unit in its area, covering the majority of the local population. It is rather interesting but not surprising [23] that the citizens in the urban area who visited HCOV, did it again for about 2 times during a one-year period; indeed, the higher contact/patient/year ratio compared to HCNM probably indicates a higher level of satisfaction with the services provided. Another interesting result is the monthly increase in HCOV users during the last year in contradistinction to HCNM, where a peak of visits is described during the summer period. This phenomenon could be attributed to the fact that urban citizens were not familiar with public PHC services and after a short period of "exploration" were increasingly visiting the HCOV.

Since the HCOV is not far away from secondary and tertiary care hospitals, the referrals of patients were expected to be more frequent than in the rural health centre [24]. This was not the case, however, as the referral rate was significantly lower. The probable self-referral of some patients could not be measured, if it actually happened, and would not be taken into consideration, since the referral rates shown in the results were exclusively based on the data coming from the Health Centres. The presence of more doctors of other than GP medical specialty in the HCNM should reduce its referral rates, considering the greater "freedom" in performing medical interventions these doctors are given by the Greek legislation compared to GPs. A possible explanation for this difference in referral rates could be the better scientific level of the doctors of HCOV due to the continuing medical education courses conducted in it and the amount of the scientific work produced.

The distribution of the diagnoses made during the study period was not similar in the two regions. The diseases of respiratory and musculoskeletal system and injuries were more frequent in the rural than in the urban area. This could be explained by the higher prevalence of smoking [25] and by heavier work and higher rural exposure to hazardous farm machinery, firearms, and open areas of water [26]. On the other hand, diseases of the cardiovascular system and endocrine, metabolic and nutritional disorders were more prevalent in the urban setting, reflecting the higher prevalence of the metabolic syndrome in metropolitan areas [27]. The low frequency of cases related with pregnancy, childbearing and family planning in both health centres might indicate the lack of confidence of patients towards the GP regarding these health conditions and it is an area of further investigation.

Asthma in childhood is more prevalent in the urban area. This could be attributed to several factors such as higher exposure to house dust mite [28] and lifestyle [29]. The prevalence of hypertension is lower in elderly rural residents. This finding is contradicted by a recent large-scale national study [30], where it is described that the control of hypertension is more effective among urban residents, partially explaining this difference.

Like other European Union countries, many people from Eastern European countries and Asia have immigrated to Greece during the last 15 years [31]. These immigrants rarely are insured and live mainly in the major cities. This situation is reflected upon the significantly higher rates of foreigners and uninsured individuals seeking care in HCOV.

Based on the PHC users' opinions, there seems to be a qualitative difference between HCOV and HCNM regarding the reason they chose the Health Centre. In contrast to the Scottish experience [32], urban residents were far more satisfied with the PHC services provided than those in Nea Madytos in all age categories, even in the adjusted samples. Moreover, proximity to their residence, low waiting time and free services were crucial subjects for the city residents. On the other hand, the uniqueness of the rural health centre as a PHC services provider and the lack of perception of other health care units lead the rural residents to HCNM. This deviation, which is supported by the constant increase in the citizens visiting the HCOV, might be explained by several factors, such as better PHC services provided, the lack of any similar unit in a city in the past, the ease of access (close to the citizens, short waiting time) and regular doctors (not usual in secondary and tertiary care hospitals).

The introduction and operation of HCOV changed the local health map in terms of providing health services to people who (more than 60%) used to visit private health care units, such as medical offices or hospitals, in their majority. Given the fact that all services in the HCOV are totally free, regardless insurance status of the patient, it can be assumed that the services provided were at least financially beneficial for the local society.

This study has some limitations. The sample of the study cannot be characterized as representative for urban and rural areas. The Health centre of Vyronas was established in 2004, compared to HCNM which is in operation since 1986. The populations the Health centres were responsible for cannot be exactly defined. General Practice/Family medicine is a new medical specialty in Greece [16,17] and the specialized doctors face many problems, such as scepticism from the patients.

The population an urban health centre has different health needs than in rural areas. On one hand, the former have more choices for medical care (public hospitals, private clinics, super-specialized medical doctors), without any need for a GP referral. On the other hand, it is easy for them to access any the medical care provider of their choice, something that is not the case in the countryside. According to the behavioural model as presented by Ronald M. Andersen [33], there are differences between those two populations regarding the potential access to medical care (presence of enabling health resources) and the actual use of those services (as derived from the results of this study and elsewhere [34]). In addition, urban and rural populations differ with regard to demographic characteristics. Given the lack of information the urban resident has regarding General Practice/Family Medicine in Greece, it is not exaggerative to say that the urban setting is a "hostile" environment for a new PHC unit.

## Conclusion

According to the results of our study, it seems that there are differences between an urban and a rural population in Greece regarding demographics, health needs and reasons for choosing a PHC unit. The aforementioned differences require a flexible health system that is able to provide the proper PHC services in each population in order to satisfy their special health needs.

Although it may be early for final conclusions, it seems that the "experiment" of introducing and operating a Primary Health Care unit in an urban setting produced some encouraging results in terms of provision of free public services that did not exist in the past. The patients' satisfaction, at least as it reported, is another hopeful fact. It is suggested that the differences found in the profile and the health needs of the citizens using PHC services between an urban and a rural area in Greece should be considered before the establishment of other PHC units in major cities.

## Competing interests

The authors declare that they have no competing interests.

## Authors' contributions

AM conceived the idea of the study, participated in study design and organized both HCOV and HCNM for the purposes of the study. CM participated in study design and performed the statistical analysis. AA was the designer of the questionnaire and its digital materialization. KM and MP were the coordinators of the data collection for both Health Centres. TM–S was responsible for the training of the participating personnel. VG performed the pilot study and participated to the randomization. DK was the coordinator for HCNM while BPM participated in study design, coordination and realization of the study.

## Pre-publication history

The pre-publication history for this paper can be accessed here:


